# On Hardy-Pachpatte-Copson's Inequalities

**DOI:** 10.1155/2014/607347

**Published:** 2014-03-18

**Authors:** Chang-Jian Zhao, Wing-Sum Cheung

**Affiliations:** ^1^Department of Mathematics, China Jiliang University, Hangzhou 310018, China; ^2^Department of Mathematics, The University of Hong Kong, Pokfulam Road, Hong Kong

## Abstract

We establish new inequalities similar to Hardy-Pachpatte-Copson's type inequalities. These results in special cases yield some of the recent results.

## 1. Introduction

The classical Hardy's integral inequality is as follows.

**Theorem A**

If *p* > 1, *f*(*x*) ≥ 0 for 0 < *x* < *∞*, and *F*(*x*) = (1/*x*)∫_0_^*x*^ *f*(*t*)*dt*, then

(1) $$\begin{array}{c} \overset{\infty }{\underset{0}{\int }}F{\left(x\right)}^{p}dx<{\left(\frac{p}{p-1}\right)}^{p}\overset{\infty }{\underset{0}{\int }}f{\left(x\right)}^{p}dx, \end{array}$$

unless *f* ≡ 0. The constant is the best possible.

Theorem A was first proved by Hardy [1], in an attempt to give a simple proof of Hilbert's double series theorem (see [2]). One of the best known and interesting generalization of the inequality (1) given by Hardy [3] himself can be stated as follows.

**Theorem B**

If *p* > 1, *m* ≠ 1,*f*(*x*) ≥ 0 for 0 < *x* < *∞*, and *F*(*x*) is defined by

(2) $$\begin{array}{c} F\left(x\right)=\overset{x}{\underset{0}{\int }}f\left(t\right)dt,\;m>1;\\ F\left(x\right)=\overset{\infty }{\underset{x}{\int }}f\left(t\right)dt,\;m<1, \end{array}$$

then

(3) $$\begin{array}{c} \overset{\infty }{\underset{0}{\int }}x^{-m}F{\left(x\right)}^{p}dx<{\left(\frac{p}{\left|m-1\right|}\right)}^{p}\overset{\infty }{\underset{0}{\int }}x^{p-m}f{\left(x\right)}^{p}dx, \end{array}$$

unless *f* ≡ 0. The constant is the best possible.

Inequalities (1) and (3) which later went by the name of Hardy's inequalities led to a great many papers dealing with alternative proofs, various generalizations, and numerous variants and applications in analysis (see [4–15]). In particular, Pachpatte [4] established some generalizations of Hardy inequalities (1) and (3). Very recently, Leng and Feng [16] proved some new Hardy-type integral inequalities. In the present paper we establish new inequalities similar to Hardy's integral inequalities (1) and (3). These results provide some new estimates to these types of inequalities and in special cases yield some of the recent results.

## 2. Main Results

Our main results are given in the following theorems.

**Theorem**

Let *a* < *b* < *R*, *c* < *d* < *R*′, *p* > 1, *q* < 1, and *α* > 0 be constants. Let *w*(*x*, *y*) be positive and locally absolutely continuous in (*a*, *b*)×(*c*, *d*). Let *h*(*x*, *y*) be a positive continuous function and let *H*(*x*, *y*) = ∫_*a*_^*x*^∫_*c*_^*y*^ *h*(*s*, *t*)*ds* *dt*, for (*x*, *y*)∈(*a*, *b*)×(*c*, *d*). Let *f*(*x*, *y*) be nonnegative and measurable on (*a*, *b*)×(*c*, *d*). If

(4) D(x,y) =1−11−qH(x,y)h(x,y)1w(x,y)∂w(x,y)∂xlog⁡(H(R,R′)H(x,y))  +p1−qH(x,y)h(x,y)×1r(x,y)∂r(x,y)∂xlog⁡(H(R,R′)H(x,y)) ≥1α,

for almost all (*x*, *y*) ∈ (*a*, *b*) × (*c*, *d*), and if *F*(*x*, *y*) is defined by

(5) $$\begin{array}{c} F\left(x,y\right)=\frac{1}{r\left(x,y\right)}\overset{x}{\underset{a}{\int }}\overset{y}{\underset{c}{\int }}r\left(s,t\right)h\left(s,t\right)f\left(s,t\right)ds\;dt \end{array}$$

for (*x*, *y*)∈(*a*, *b*)×(*c*, *d*), then

(6) ∫cd∫abw(x,y)H(x,y)−1h¯(x,y)   ×(log⁡(H(R,R′)H(x,y)))−qF(x,y)pdx dy ≤[α(p1−q)]p  ×∫cd∫ab[(log⁡(H(R,R′)H(x,y)))p−qw(x,y)H(x,y)p−1×  h(x,y)1−pG(x,y)p]dx dy,

where

(7) $$\begin{array}{c} \bar{h}\left(x,y\right)=\overset{y}{\underset{c}{\int }}h\left(x,t\right)dt,\\ G\left(x,y\right)=\frac{1}{r\left(x,y\right)}\overset{y}{\underset{c}{\int }}r\left(x,t\right)h\left(x,t\right)f\left(x,t\right)dt. \end{array}$$

**Remark 2**

Let *f*(*x*, *y*), *w*(*x*, *y*), *h*(*x*, *y*), and *r*(*x*, *y*) reduce to *f*(*x*), *w*(*x*), *h*(*x*), and *r*(*x*), respectively, and with suitable modifications in Theorem 1, (6) changes to the following result:

(8) ∫abw(x)H(x)−1h(x)(log⁡(H(R)H(x)))−qF(x)pdx ≤[α(p1−q)]p  ×∫ab[(log⁡(H(R)H(x)))p−q×w(x)H(x)p−1h(x)f(x)p]dx.

This is just a new inequality established by Pachpatte [4].

Moreover, we note that the inequality established in Theorem 1 is the further generalizations of the inequality established by Copson [17].

Taking for *w*(*x*) = *r*(*x*) = 1,  *H*(*R*) = *R*, and *α* = 1 in (8), (8) changes to the following result:

(9) ∫abH(x)−1h(x)(log⁡(RH(x)))−qF(x)pdx ≤(p1−q)p  ×∫ab[(log⁡(RH(x)))p−qH(x)p−1h(x)f(x)p]dx.

This is just a new inequality established by Love [7].

Let *h*(*x*) = 1, *a* → 0,  *b* → *∞*, and log⁡ (*R*/(*x* − *a*)) = 1 in (9); then (9) changes to the following result:

(10) $$\begin{array}{c} \overset{\infty }{\underset{0}{\int }}x^{-1}F{\left(x\right)}^{p}dx\leq {\left(\frac{p}{1-q}\right)}^{p}\overset{\infty }{\underset{0}{\int }}x^{p-1}f{\left(x\right)}^{p}dx. \end{array}$$

This result is obtained in (3) stated in the Introduction.

**Theorem 3**

Let *a* < *b* < *R*, *c* < *d* < *R*′, *p* > 1, *q* > 1, and *β* > 0 be constants. Let *w*(*x*, *y*) be positive and locally absolutely continuous in (*a*, *b*)×(*c*, *d*). Let *h*(*x*, *y*) be a positive continuous function and let *H*(*x*, *y*) = ∫_*a*_^*x*^∫_*c*_^*y*^ *h*(*s*, *t*)*ds* *dt*, for (*x*, *y*)∈(*a*, *b*)×(*c*, *d*). Let *f*(*x*, *y*) be nonnegative and measurable on (*a*, *b*)×(*c*, *d*). Let

(11) E(x,y) =1−1q−1H(x,y)h(x,y)1w(x,y)∂w(x,y)∂ylog⁡(H(R,R′)H(x,y))  +pq−1H(x,y)h(x,y)×1r(x,y)∂r(x,y)∂ylog⁡(H(R,R′)H(x,y)) ≥1β,

for almost all (*x*, *y*)∈(*a*, *b*)×(*c*, *d*). If *F*(*x*, y) is defined by

(12) $$\begin{array}{c} F\left(x,y\right)=\frac{1}{r\left(x,y\right)}\overset{b}{\underset{x}{\int }}\overset{d}{\underset{y}{\int }}r\left(s,t\right)h\left(s,t\right)f\left(s,t\right)ds\;dt \end{array}$$

for (*x*, *y*)∈(*a*, *b*)×(*c*, *d*), then

(13) ∫cd∫abw(x,y)H(x,y)−1h~(x,y)   ×(log⁡(H(R,R′)H(x,y)))−qF(x,y)pdx dy ≤[β(pq−1)]p  ×∫cd∫ab[(log⁡(H(R,R′)H(x,y)))p−qw(x,y)H(x,y)p−1×  h(x,y)1−pL(x,y)p]dx dy,

where

(14) $$\begin{array}{c} \tilde{h}\left(x,y\right)=\overset{x}{\underset{a}{\int }}h\left(s,y\right)ds, \end{array}$$

**Remark 4**

Let *f*(*x*, *y*), *w*(*x*, *y*), *h*(*x*, *y*), and *r*(*x*, *y*) reduce to *f*(*x*), *w*(*x*), *h*(*x*), and *r*(*x*), respectively, and with suitable modifications in Theorem 3, (13) changes to the following result:

(15) ∫abw(x)H(x)−1h(x)(log⁡(H(R)H(x)))−qF(x)pdx ≤[β(pq−1)]p∫ab[(log⁡(H(R)H(x)))p−q×  w(x)H(x)p−1h(x)f(x)p]dx.

This is just a new inequality established by Pachpatte [4].

On the other hand, we note that the inequality established in Theorem 3 is the further generalizations of the inequality established by Copson [17].

Taking for *w*(*x*) = *r*(*x*) = 1, *H*(*R*) = *R*, and *β* = 1 in (15), (15) changes to the following result:

(16) ∫abH(x)−1h(x)(log⁡(RH(x)))−qF(x)pdx ≤(pq−1)p∫ab[(log⁡(RH(x)))p−q×  H(x)p−1h(x)f(x)p]dx.

This is just a new inequality established by Love [7].

## 3. Proof of Theorems

**Proof of Theorem 1**

If we let *u*(*x*, *y*) = *w*(*x*, *y*)*F*(*x*, *y*)^*p*^ and in view of

(17) $$\begin{array}{c} F\left(x,y\right)=\frac{1}{r\left(x,y\right)}\overset{x}{\underset{a}{\int }}\overset{y}{\underset{c}{\int }}r\left(s,t\right)h\left(s,t\right)f\left(s,t\right)ds\;dt \end{array}$$

for (*x*, *y*)∈(*a*, *b*)×(*c*, *d*), then

(18) ∂u(x,y)∂x =∂w(x,y)∂xF(x,y)p+w(x,y)pF(x,y)p−1  ×(1r(x,y)∫cyr(x,t)h(x,t)f(x,t)dt−∂r(x,y)/∂xr2(x,y)×∫ax∫cyr(s,t)h(s,t)f(s,t)ds dt).

Let

(19) $$\begin{array}{c} \frac{\partial v\left(x,y\right)}{\partial x}=H{\left(x,y\right)}^{-1}\bar{h}\left(x,y\right){\left[\log\left(\frac{H\left(R,R^{\prime }\right)}{H\left(x,y\right)}\right)\right]}^{-q}, \end{array}$$

where $\bar{h}(x,y)=\int_{c}^{y}h(x,t)dt$ and in view of *H*(*x*, *y*) = ∫_*a*_^*x*^∫_*c*_^*y*^ *h*(*s*, *t*)*ds* *dt*, for (*x*, *y*)∈(*a*, *b*)×(*c*, *d*), then

(20) $$\begin{array}{c} v\left(x,y\right)=-\frac{{\left[\log\left(H\left(R,R^{\prime }\right)/H\left(x,y\right)\right)\right]}^{-q+1}}{\left(-q+1\right)}. \end{array}$$

From (18), (20), and integrating by parts for *x*, we have

(21) ∫cd∫abw(x,y)H(x,y)−1h¯(x,y)  ×(log⁡(H(R,R′)H(x,y)))−qF(x,y)pdx dy =−∫cd{w(x,y)F(x,y)p×[log⁡(H(R,R′)/H(x,y))]−q+1−q+1|x=ax=b−∫ab[log⁡(H(R,R′)/H(x,y))]−q+1−q+1×[∂w(x,y)∂xF(x,y)p+w(x,y)pF(x,y)p−1×(G(x,y)−∂r(x,y)/∂xr2(x,y)×∫ax∫cyr(s,t)h(s,t)× f(s,t)ds dt)]dx}dy,

where

(22) $$\begin{array}{c} G\left(x,y\right)=\frac{1}{r\left(x,y\right)}\overset{y}{\underset{c}{\int }}r\left(x,t\right)h\left(x,t\right)f\left(x,t\right)dt. \end{array}$$

If *q* < 1, then we observe that

(23) ∫cd∫abD(x,y)w(x,y)H(x,y)−1h¯(x,y)   ×(log⁡(H(R,R′)H(x,y)))−qF(x,y)pdx dy ≤p1−q∫cd∫ab[w(x,y)(log⁡(H(R,R′)H(x,y)))−q+1×  G(x,y)F(x,y)p−1]dx dy =p1−q  ×∫cd∫ab[{(log⁡(H(R,R′)H(x,y)))−q}1/p×log⁡(H(R,R′)H(x,y))w(x,y)1/p×H(x,y)(p−1)/p       ×  h(x,y)−(p−1)/pG(x,y)]     ×[{(log⁡(H(R,R′)H(x,y)))−q}(p−1)/p       ×w(x,y)(p−1)/p       ×  [H(x,y)−1×h(x,y)](p−1)/p       ×  F(x,y)p−1]dx dy.

By applying Hölder's inequality with indices *p*, *p*/(*p* − 1) on the right side of (23), we obtain

(24) ∫cd∫abw(x,y)H(x,y)−1h¯(x,y)   ×(log⁡(H(R,R′)H(x,y)))−qF(x,y)pdx dy ≤α(p1−q)  ×{∫cd∫ab[(log⁡(H(R,R′)H(x,y)))−q       ×(log⁡(H(R,R′)H(x,y)))p       ×w(x,y)H(x,y)p−1       ×  h(x,y)−(p−1)G(x,y)p]dx dy}1/p  ×{∫cd∫ab[(log⁡(H(R,R′)H(x,y)))−qw(x,y)H(x,y)−1×  h(x,y)F(x,y)p]dx dy}(p−1)/p.

Dividing both sides of (24) by the second integral factor on the right side of (24) and raising both sides to the *p*th power, we obtain

(25) ∫cd∫abw(x,y)H(x,y)−1h¯(x,y)   ×(log⁡(H(R,R′)H(x,y)))−qF(x,y)pdx dy ≤[α(p1−q)]p  ×∫cd∫ab[(log⁡(H(R,R′)H(x,y)))p−q×w(x,y)H(x,y)p−1×  h(x,y)−(p−1)G(x,y)p]dx dy.

**Proof of Theorem 3**

If we let *u*(*x*, *y*) = *w*(*x*, *y*)*F*(*x*, *y*)^*p*^ and in view of

(26) $$\begin{array}{c} F\left(x,y\right)=\frac{1}{r\left(x,y\right)}\overset{b}{\underset{x}{\int }}\overset{d}{\underset{y}{\int }}r\left(s,t\right)h\left(s,t\right)f\left(s,t\right)ds\;dt \end{array}$$

for (*x*, *y*)∈(*a*, *b*)×(*c*, *d*), then

(27) ∂u(x,y)∂y =∂w(x,y)∂yF(x,y)p+w(x,y)pF(x,y)p−1  ×(−1r(x,y)∫ydr(x,t)h(x,t)f(x,t)dt−∂r(x,y)/∂yr2(x,y)∫xb∫ydr(s,t)h(s,t)f(s,t)ds dt).

Let

(28) $$\begin{array}{c} \frac{\partial v\left(x,y\right)}{\partial y}=H{\left(x,y\right)}^{-1}\tilde{h}\left(x,y\right){\left[\log\left(\frac{H\left(R,R^{\prime }\right)}{H\left(x,y\right)}\right)\right]}^{-q}, \end{array}$$

where $\tilde{h}(x,y)=\int_{a}^{x}h(s,y)ds$ and in view of *H*(*x*, *y*) = ∫_*a*_^*x*^∫_*c*_^*y*^ *h*(*s*, *t*)*ds* *dt*, for (*x*, *y*)∈(*a*, *b*)×(*c*, *d*), then

(29) $$\begin{array}{c} v\left(x,y\right)=-\frac{{\left[\log\left(H\left(R,R^{\prime }\right)/H\left(x,y\right)\right)\right]}^{-q+1}}{\left(-q+1\right)}. \end{array}$$

From (27), (29), and integrating by parts for *y*, we have

(30) ∫cd∫abw(x,y)H(x,y)−1h~(x,y)   ×(log⁡(H(R,R′)H(x,y)))−qF(x,y)pdx dy =−∫ab{w(x,y)F(x,y)p×[log⁡(H(R,R′)/H(x,y))]−q+1−q+1|y=cy=d−∫cd[log⁡(H(R,R′)/H(x,y))]−q+1−q+1×[∂w(x,y)∂yF(x,y)p+w(x,y)pF(x,y)p−1×(L(x,y)−∂r(x,y)∂yr2(x,y)×∫xb∫ydr(s,t)h(s,t)× f(s,t)ds dt)]dy}dx,

where

(31) $$\begin{array}{c} L\left(x,y\right)=-\frac{1}{r\left(x,y\right)}\overset{b}{\underset{x}{\int }}r\left(s,y\right)h\left(s,y\right)f\left(s,y\right)ds. \end{array}$$

If *q* > 1, then we observe that

(32) ∫cd∫abE(x,y)w(x,y)H(x,y)−1h~(x,y)   ×(log⁡(H(R,R′)H(x,y)))−qF(x,y)pdx dy ≤pq−1∫cd∫ab[w(x,y)(log⁡(H(R,R′)H(x,y)))−q+1×  L(x,y)F(x,y)p−1]dx dy =pq−1  ×∫cd∫ab[{(log⁡(H(R,R′)H(x,y)))−q}1/p×log⁡(H(R,R′)H(x,y))w(x,y)1/pH(x,y)(p−1)/p×  h(x,y)−(p−1)/pL(x,y)]  ×[{(log⁡(H(R,R′)H(x,y)))−q}(p−1)/p×w(x,y)(p−1)/p×[H(x,y)−1×h(x,y)](p−1)/p×  F(x,y)p−1]dx dy.

By applying Hölder's inequality with indices *p*, *p*/(*p* − 1) on the right side of (32), we obtain

(33) ∫cd∫abw(x,y)H(x,y)−1h~(x,y)   ×(log⁡(H(R,R′)H(x,y)))−qF(x,y)pdx dy ≤β(pq−1)  ×{∫cd∫ab[(log⁡(H(R,R′)H(x,y)))−q×(log⁡(H(R,R′)H(x,y)))pw(x,y)H(x,y)p−1×  h(x,y)−(p−1)L(x,y)p]dx dy}1/p  ×{∫cd∫ab[(log⁡(H(R,R′)H(x,y)))−q×w(x,y)H(x,y)−1×  h(x,y)F(x,y)p]dx dy}(p−1)/p.

Dividing both sides of (33) by the second integral factor on the right side of (33) and raising both sides to the *p*th power, we obtain

(34) ∫cd∫abw(x,y)H(x,y)−1h~(x,y)   ×(log⁡(H(R,R′)H(x,y)))−q×F(x,y)pdx dy ≤[β(pq−1)]p  ×∫cd∫ab[(log⁡(H(R,R′)H(x,y)))p−qw(x,y)H(x,y)p−1×  h(x,y)−(p−1)L(x,y)p]dx dy.
